# Effect of host switching simulation on the fitness of the gregarious parasitoid *Anaphes flavipes* from a novel two-generation approach

**DOI:** 10.1038/s41598-021-98393-y

**Published:** 2021-09-30

**Authors:** Alena Samková, Jan Raška, Jiří Hadrava, Jiří Skuhrovec

**Affiliations:** 1 Department of Plant Protection, Faculty of Agrobiology, Food and Natural Resources, Czech University of Life Sciences Prague, Kamýcká 129, 165 00 Prague 6-Suchdol, Czech Republic; 2 Department of Zoology, Faculty of Science, Charles University, Viničná 7, 128 43 Prague 2, Czech Republic; 3 Institute of Entomology, Biological Centre, Czech Academy of Sciences, Branišovská 31, 370 05 České Budějovice, Czech Republic; 4 Crop Research Institute, Drnovská 507, 161 06 Praha 6-Ruzyně, Czech Republic

**Keywords:** Ecology, Zoology

## Abstract

Herbivorous insects can escape the strong pressure of parasitoids by switching to feeding on new host plants. Parasitoids can adapt to this change but at the cost of changing their preferences and performance. For gregarious parasitoids, fitness changes are not always observable in the F1 generation but only in the F2 generation. Here, with the model species and gregarious parasitoid *Anaphes flavipes,* we examined fitness changes in the F1 generation under pressure from the simulation of host switching, and by a new two-generation approach, we determined the impact of these changes on fitness in the F2 generation. We showed that the parasitoid preference for host plants depends on hatched or oviposited learning in relation to the possibility of parasitoid decisions between different host plants. Interestingly, we showed that after simulation of parasitoids following host switching, in the new environment of a fictitious host plant, parasitoids reduced the fictitious host. At the same time, parasitoids also reduced fertility because in fictitious hosts, they are not able to complete larval development. However, from a two-generation approach, the distribution of parasitoid offspring into both native and fictitious hosts caused lower parasitoid clutch size in native hosts and higher individual offspring fertility in the F2 generation.

## Introduction

Natural enemies keep herbivores at densities lower than those that would deplete host plants^1^. Parasitoids are important natural enemies that maintain the natural balance of ecosystems^2,3^, with all insects in a given habitat potentially being hosts for parasitoids^4^. However, herbivorous insects, as the most vulnerable hosts^4^, are not passive participants; they evolve mechanical, physiological and immune defences against parasitoids^2^ and are in a constant evolutionary arms race. Regardless of the defensive mechanisms the host invents, parasitoids evolve to overcome them^5,6^.

In addition to direct defence, herbivorous insects can escape parasitoids by switching to a novel host plant and obtaining enemy free space^7^. Herbivores usually experience lower performance when colonizing a novel host plant^8^, but the associated fitness reduction may be compensated by lower predation and parasitism on this plant^9,10^. Although locating hosts in a new microhabitat is not easy for parasitoids, there are known cases of this switching^11,12^. These new habitats pose several challenges for parasitoids^13^, that might reduce their fitness, either directly (e.g. encounter of a plant toxin or metabolite by the juvenile stage) or indirectly (e.g. effect of decreased host size and quality on the parasitoid)^14^. In addition to possible changes in parasitoid fitness related to the new host plant of their host^8,15^, the new environment may present not only new predators of the parasitized hosts^16^ but also new potential host species for parasitoids^17^.

In this changing environment, it is critical whether the parasitoid’s reproductive strategy is solitary or gregarious: solitary parasitoids respond to these changes primarily by choosing the sex of the offspring developing in the host^18^, whereas gregarious parasitoids can choose both sex ratio and clutch size^19,20^. Female solitary parasitoids usually lay one egg or sometimes multiple eggs in a single host, but only one offspring completes development. In contrast, multiple offspring may complete development within a single host in gregarious parasitoids^21,22^. In parasitoids, the gregarious strategy has evolved numerous times (independently at least 43 times in 26 different families of Hymenoptera) from the ancestral solitary strategy^23^. The emergence of the gregarious strategy has been explained by “gregariousness based on non-fighting larval phenotypes”^24^, where loss of predatory larvae led to the origin of the gregarious strategy under very strict conditions^25–27^. Under this assumption, however, any interactions between solitary and gregarious larvae would result in a highly asymmetric result^24^, because when sharing the same host, tolerant gregarious larvae would be killed by predatory solitary larvae^26^. Thus, newer theoretical models demonstrate a much more likely evolution of gregarious strategy based on larval mobility, "gregariousness based on non-searching larval phenotypes", rather than on the lack of fighting larvae reviewed by^24,25^. For example, Mayhew & van Alphen^28^ show two closely related species with predatory larvae with solitary (*Aphaereta genevensis* Fischer, 1966) and gregarious (*Aphaereta pallipes* (Say, 1829)) strategies, where the results of superparasitization showed that the gregarious strategy is possible by differences in larval behaviour. Pexton & Mayhew^29^ report that there is a study of Rosenheim^27^ where the origin of a gregarious strategy depends on the larval tolerance, in the context of same-sex offspring developing in the same host, as female offspring are more closely related in parasitoids^30^. Thus, more than one change in larval phenotype is responsible for the emergence of a gregarious strategy from a solitary one: is the loss of aggression while retaining mobility and the other is the retention of aggression but with reduced mobility^29^.

It would seem that the solitary strategy is preferable as more parasitoid species adopt this reproductive strategy^31^. However, as mentioned above, the gregarious strategy gives an advantage to its holders in the form of multiple offspring that are able to complete development in a single host. This allows gregarious parasitoids to respond flexibly to changing environmental conditions, such as different host population densities^20,32^, host characteristics^33^, or presence of a predator of host^20^. For example, the population density of solitary parasitoids decreases with lower host densities, because only one individual develops in one host^34^, whereas gregarious parasitoids increase clutch size at lower host densities and thus maintain stable population density^20^. However, females of gregarious parasitoids must be able to choose the suitable clutch size not only according to environmental conditions^35^, but also according to whether their host does not accept food after parasitization (idiobiont) or whether it continues accepting food, increasing its the body size and the amount of resources for the parasitoid's offspring (koinobiont)^36^. In some cases of hemolymph-feeding koinobionts, such as species of genera *Cotesia* Cameron, 1891 and *Microplitis* Foerster, 1862, large part of the host remains intact after the parasitoid hatches^37^. These solitary parasitoids can evolve gregariously provided they have sufficient host resources to develop offspring without reducing their fitness^36^.

For both koinobionts and idiobionts, the change in clutch size, largely affect offspring body sizes, because the body size of the offspring in many cases depends on the amount of food obtained during larval development^23,38^ and the body size therefore decreases with higher clutch size^39–41^. At the same time, the offspring body size determines their future fertility in the F2 generation^40–42^. In general, the population density not only of parasitoids but also of other insects is determined by the number of individuals of a given species, i.e. it can be predicted from the fertility of females^43,44^. For gregarious parasitoids, a two-generation approach has been proposed, which provides more accurate results about parasitoid population dynamics by involving changes in fertility in the F1 and F2 generations due to different clutch sizes^45^.

In this study, using a two-generation approach, we examine the response of the gregarious parasitoid *Anaphes flavipes* (Förster, 1841) (Hymenoptera: Mymaridae) to the simulation of host switching to a fictitious host plant. Using a two-generation approach, we determined the consequences of changes in clutch size on *A. flavipes* fitness in the F2 generation. The idiobiont gregarious parasitoid *A. flavipes* is a suitable model for studying these questions because mated females parasitize the host in the egg stage, which represents the ultimate food supply for their offspring have a choice of 35 possible clutch size combinations (1 to 7 offspring laid in one host in any sex ratio)^41,46^. Using a two-generation approach, some combinations of clutch size are more advantageous than others^47,48^ because they ensure higher fertility in the F2 generation^45^. In addition, knowledge of the factors that affect the fertility and thus the population dynamics of *A. flavipes* will be helpful in the use of this wasp in biological control against economically important pests of the genus *Oulema*^49,50^.

## Result

### No-choice test of host plants

The offspring sex ratio was not affected by the substrate on which host eggs were offered for parasitization (usual host plant versus filter paper: GLMM-b, χ^2^_(1)_ = 0.017, p = 0.896; fictitious host plant versus filter paper: GLMM-b, χ^2^_(1)_ = 0.618, p = 0.432; fictitious host plant versus usual host plant GLMM-b, χ^2^_(1)_ = 0.954, p = 0.329). The proportion of parasitized hosts was higher on filter paper than on usual host plants (GLM-b, χ^2^_(1)_ = 13.457, p < 0.001) and fictitious host plants (GLM-b, χ^2^_(1)_ = 8.752, p = 0.003). The proportion of parasitized hosts between usual and fictitious host plants was not statistically significant (GLM-b, χ^2^_(1)_ = 0.515, p = 0.473). The clutch size was higher on the filter paper than on the fictitious host plant (GLMM-p, χ^2^_(1)_ = 3.317, p = 0.069). The clutch size between usual host plants and fictitious host plants (GLMM-p, χ^2^_(1)_ = 1.334, p = 0.248) or filter paper (GLMM-p, χ^2^_(1)_ = 0.126, p = 0.723) was not statistically significant. Overall, wasps laid more offspring on filter paper than on usual (LM, F_(1,38)_ = 5.048, p = 0.031) and fictitious (LM, F_(1,38)_ = 6.293, p = 0.017) plants. The difference between usual and fictitious plants was not significant (LM, F_(1,38)_ = 0.081, p = 0.777) (Suppl. Mat. 1).

### Choice test of host plants

The host plant in choice tests affects the reproductive characteristics of *A. flavipes*. The offspring sex ratio was not affected by the host plant (usual host plant versus filter paper: GLMM-b, χ^2^_(1)_ = 1.454, p = 0.228; fictitious host plant versus filter paper: GLMM-b, χ^2^_(1)_ = 3.611, p = 0.057; fictitious host plant versus usual host plant: GLMM-b, χ^2^_(1)_ = 0.795, p = 0.373). The proportion of parasitized hosts was higher on the usual host plant than on the fictitious host plant (GLM-b, χ^2^_(1)_ = 11.397, p = 0.001) and filter paper (GLM-b, χ^2^_(1)_ = 4.416, p = 0.036). The proportion of parasitized hosts between fictitious host plants and filter paper was not statistically significant (GLM-b, χ^2^_(1)_ = 1.452, p = 0.228). The clutch size was not statistically significant for usual host plants compared to fictitious host plants (GLMM-p, χ^2^_(1)_ = 0.103, p = 0.748) or filter paper (GLMM-p, χ^2^_(1)_ = 0.29, p = 0.591) or for fictitious host plants compared to filter paper (GLMM-p, χ^2^_(1)_ = 0.028, p = 0.867) (Suppl. Mat. 2).

### Effect of host plant choice tests versus no-choice tests

**Figure 1:**
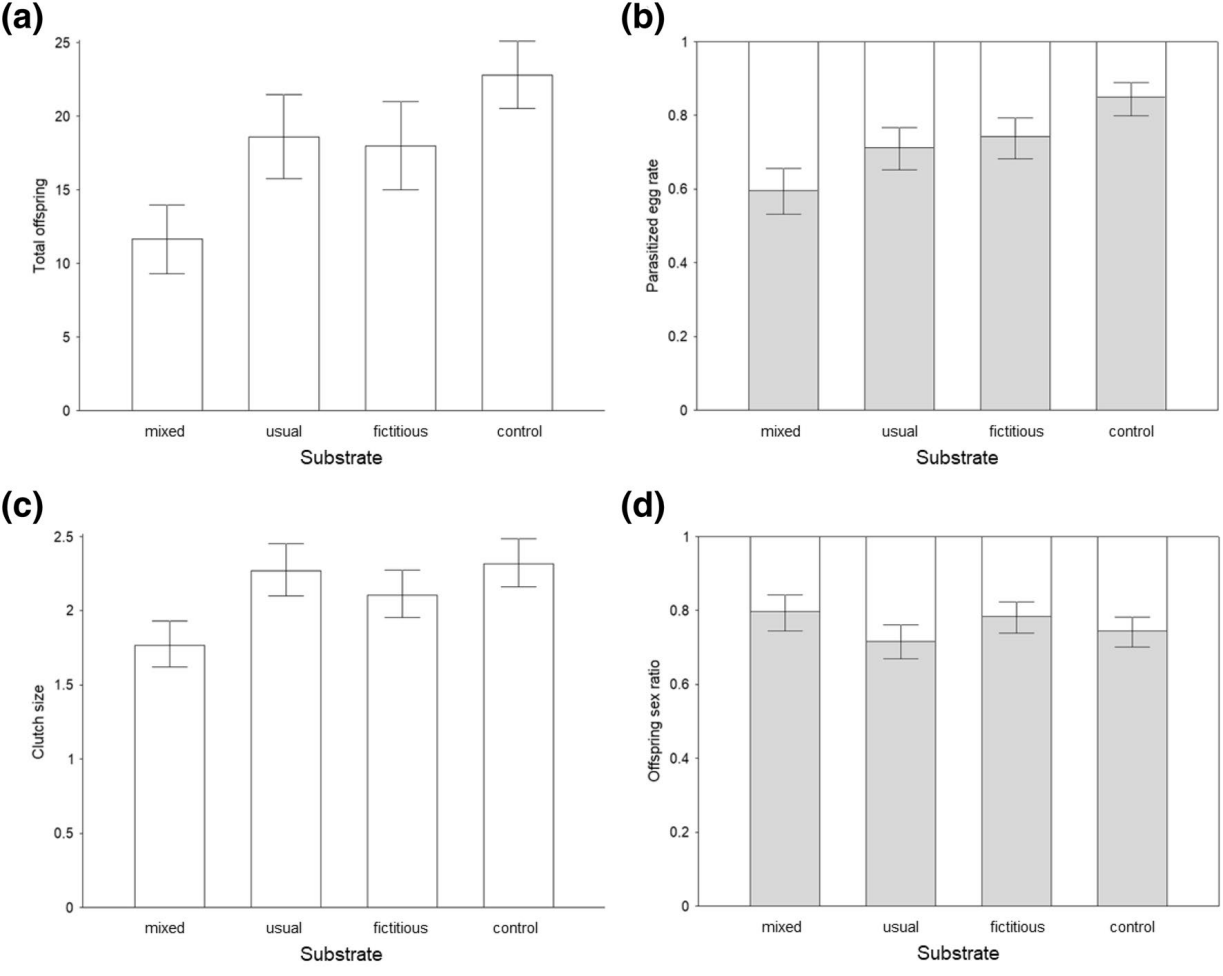
Effect of host egg substrate (mixed, usual host plant (crop), fictitious host plant (broad-leaved dock), control substrate (filter paper)) on reproductive strategy of *A. flavipes*: (**a**) offspring sex ratio (grey: females, blank: males), (**b**) parasitized host egg rate (grey: parasitized), (**c**) clutch size (i.e. offspring per parasitized host egg), and (**d**) total number of offspring per female. Mean values and 95% confidence interval.

The type of test, “no-choice” and “choice”, affected the reproductive characteristics of *A. flavipes*. The total number of offspring by one female (LM, F_(1,78)_ = 25.783, p < 0.001; Fig. 1a), the proportion of parasitized hosts (GLM-b, χ^2^_(1)_ = 25.519, p < 0.001; Fig. 1b) and clutch size (GLM-p, χ^2^_(1)_ = 19.002, p < 0.001; Fig. 1c) were higher if the wasps were hosted on one type of host plant (no-choice test) (Suppl. Mat. 1, 2). The offspring sex ratio was not affected between different types of tests (GLMM-b, χ^2^_(1)_ = 1.427, p = 0.232; Fig. 1d).

### Effect of fictitious host

**Figure 2:**
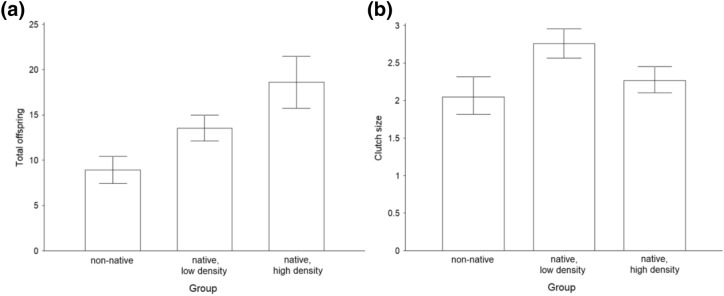
(**a**) Clutch size (i.e. offspring per parasitized host egg) and (**b**) total number of offspring per female *A. flavipes* under different conditions: fictitious host plant with 50% rate of an unsuitable host (broad-leaved dock, 6 eggs of *Oulema* sp., 6 eggs of *Gastrophysa viridula*); usual host plant, low host density (crop, 6 eggs of *Oulema* sp.); usual host plant, high host density (crop, 12 eggs of *Oulema* sp.). Mean values and 95% confidence interval.

The offspring sex ratio (GLMM-b, F_(1,25)_ = 0.448, p = 0.503) and the proportion of parasitized hosts (GLM-b, χ^2^_(1)_ = 1.282, p = 0.258) were not statistically significant between wasps with 6 fictitous and 6 native hosts and wasps with 6 native hosts offered for parasitization, but fertility (LM, df = 25, χ^2^_(1)_ = 18.676, p < 0.001; Fig. 2a) and clutch size (LMM, χ^2^_(1)_ = 16.363, p < 0.001; Fig. 2b) were higher for wasps with 6 native hosts offered for parasitization. When comparing wasps with 6 native and 6 fictitous host eggs with wasps with 12 host eggs offered for parasitization, the fertility (LM, F_(1,37)_ = 40.526, p < 0.001; Fig. 2a) was higher for wasps with 12 native host eggs offered for parasitization. The differences in offspring sex ratio (GLMM-b, χ^2^_(1)_ = 1.573, p = 0.21), the proportion of parasitized hosts (GLM-b, χ^2^_(1)_ = 0.427, p = 0.513) and clutch size (GLMM-p, χ^2^_(1)_ = 1.53, p = 0.216; Fig. 2b) were not statistically significant. The rate of emergence for fictitous hosts with parasitoids was 22%, and that for control fictitous hosts without parasitoids was 94% (Suppl. Mat. 3).

## Discussion

**Figure 3:**
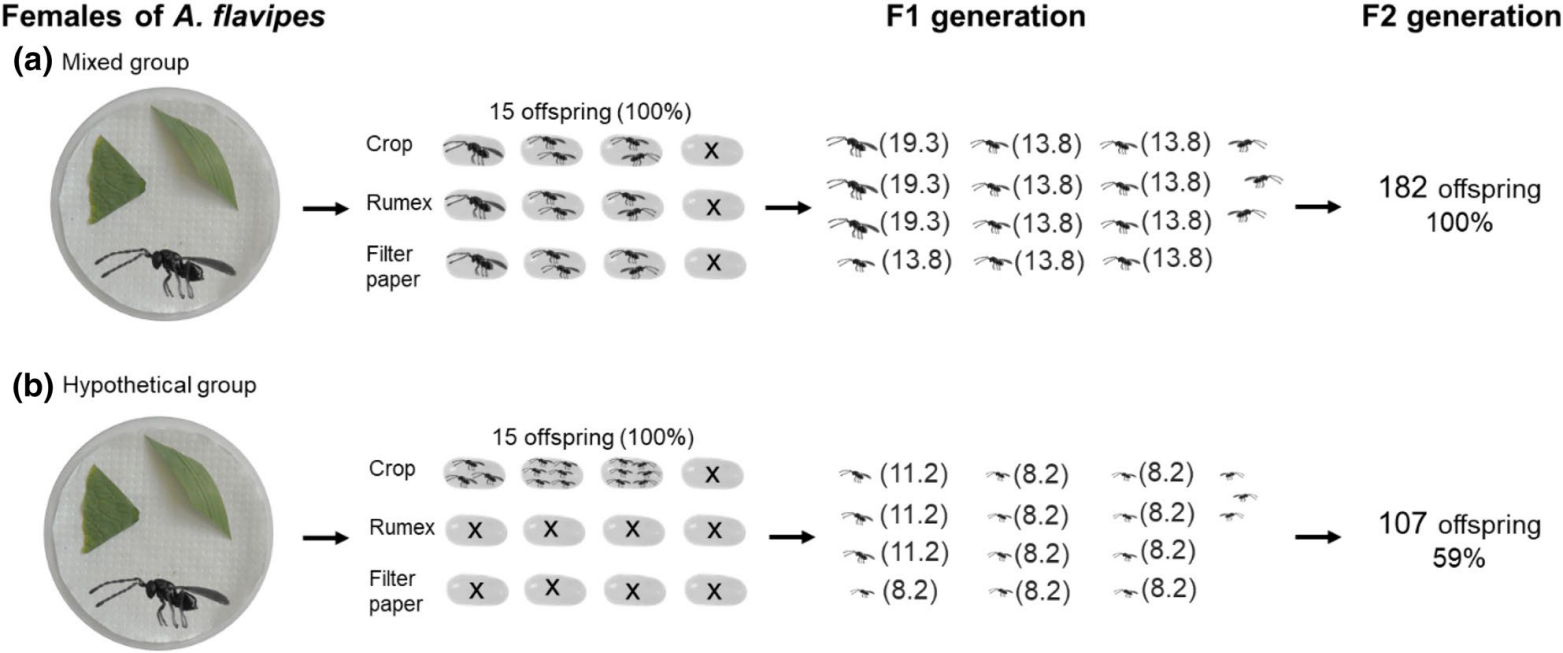
A hypothetical scenario showing the importance of a two-generation approach; the females have host eggs for parasitation offered on the usual host plant (crop), fictitious host plant (*Rumex*) and control substrate (filter paper). (**a**) the female, which accepts the all host eggs offered on the different host plans (real scenario (Suppl. Mat. 4)) and (**b**) the female, which accepts the only host eggs offered on the usual host plant may have the same fertility in F1 generation, but using different clutch size, they obtain different fertility in F2 generation (fertility was determined according to Samková et al.^45^). (*The fertility values (shown in parentheses); The offspring sex ratio of *A. flavipes* is 3:1 (male:female)^46^; females are shown as (left oriented) and males as (right oriented)).

In general, parasitoids show remarkable differences in their reproductive strategies, with their success depending on many factors such as ovigeny, fecundity, life span and many other^51^. In this study, we focused on one of the factors that affects the successful reproduction of parasitoids^52,53^, namely the localization of a suitable host in relation to a distinct host plant. A considerable number of studies show that parasitoids are influenced by plant defenses, either by changes in plant volatiles that hosts can use to defend themselves against parasitation or, conversely, these toxins degrade host quality for parasitoids^54–56^. However, there is a need to examine the effect of plants, herbivores and parasitoids in broader (“tritrophic”) context, and to uncover many direct and indirect interactions that will vary in strength and significance^57^. Thus, here, we presented the effect of host plants and the effect of a fictitious host that the parasitoid may encounter in the new environment of the fictitious plant on the fitness of parasitoids from a novel two-generation approach^45^. Using the model species *A. flavipes*, in Fig. 3, we show the hypothetical importance of this approach for studying parasitoid fitness. For example, in a situation where founders would refuse the host on the fictitious host plant, the changes in fertility in relation to a different type of host plant may not be observed in the F1 generation but will appear in the F2 generation.

**Figure 4:**
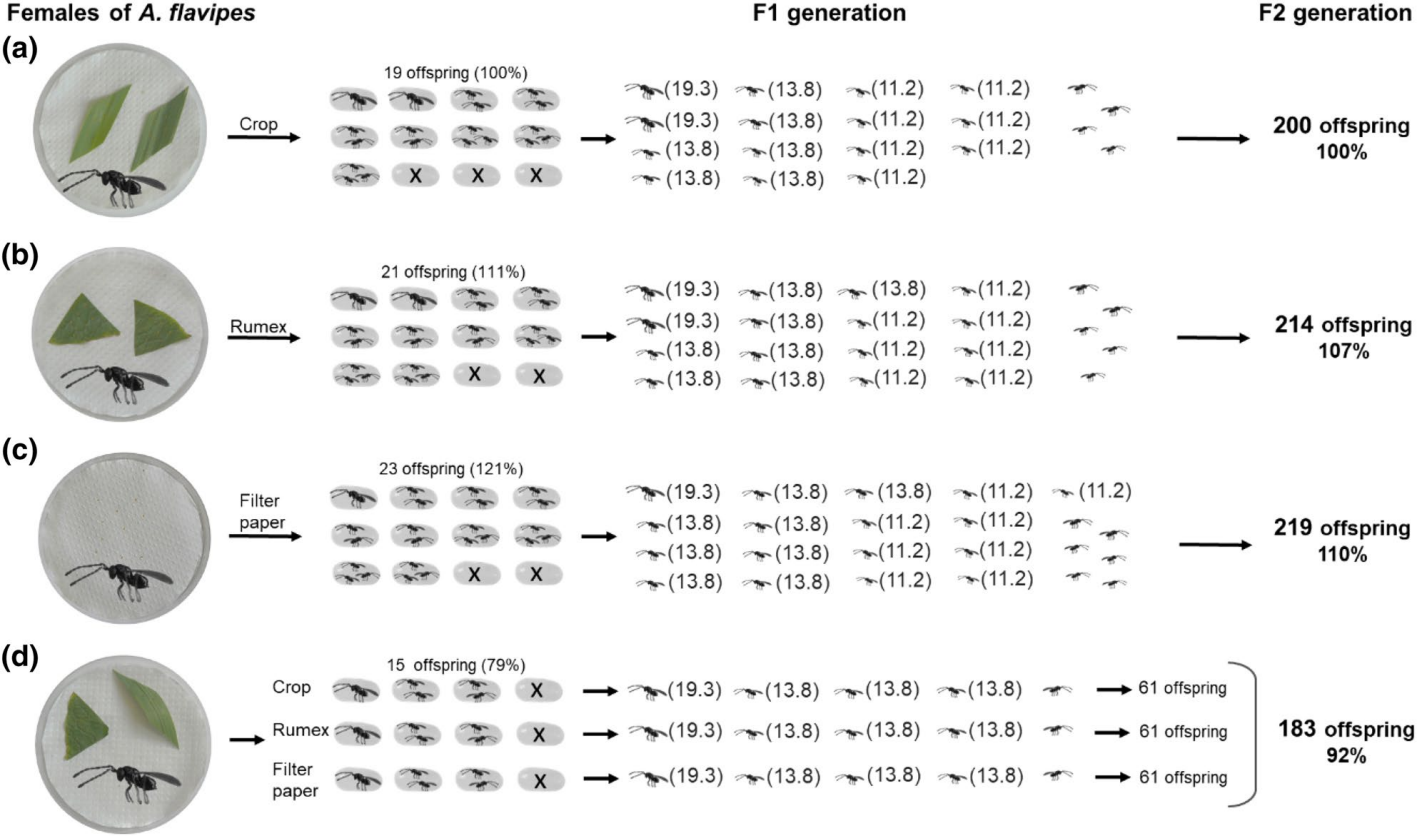
The relationship between different fitness characteristics in F1 generation and fertility in F2 generation for four groups of females with different type of substrate on which the host eggs were offered; the 12 host eggs were offered on the (1) Non-choice test: (**a**) usual host plant (crop); (**b**) fictitious host plant (*Rumex*); (**c**) control substrate (filter paper) and (2) Choice test: (**d**) combination on all (usual, fictitious host plant and control substrate). (*The fertility values (shown in parentheses); To simplify the model the male offspring are not shown).

Recognition of the host plant by the parasitoid is fixed before adult emergence^58,59^, immediately following emergence^60^, or during oviposition^61^. In our no-choice tests, females of *A. flavipes* parasitized more host eggs on the substrate on which they hatched (control substrate; Fig. 4c) than on the usual and fictitious host plant (Fig. 4a,b), which would indicate that females learn about the host plant before or shortly after hatching. This finding may also be related to the fact that *A. flavipes* females hatch with a final number of own eggs (pro-ovigeny type)^62^ that can be laid immediately after hatching^46^. Based on the fact that synovigenic species are longer lived than proovigenic species^63^, it can be assumed that synovigenic species have a longer time to recognize the host plant. However, the influence of the ovaries in relation to host plant recognition is debatable because the imprinting of the host plant after the emergence of the parasitoids and, in relation to this, their higher fitness have been confirmed in quite a number of studies^64,65^ and these studies included both synovigenic (*Trichogramma brassicae* Bezdenko, 1968)^63^ and proovigenic (*Cotesia* Cameron 1891)^63^ parasitoid species.

Interestingly, if females had a choice between the usual host plant, fictitious host plant and the control substrate, they chose to parasitize more host eggs on the usual host plant than on the learned substrate during emergence (control substrate) (Fig. 4d). This would indicate that the females can recognize the usual host plant if they have a choice between substrates, i.e., learning during oviposition^61^.

When comparing the results of the choice test and the no-choice test, females with a choice between substrates on which host eggs are offered for parasitization always have significantly lower values of fitness characteristics—the number of parasitized hosts, the number of offspring and the clutch size (Fig. 4). Although many factors are involved in fitness in the broader sense, one of the main indicators is still undoubtedly the number of offspring, especially when fitness is measured by maternal fertility according to the traditional approach^43,44^. However, the two-generation approach involves a change in fertility between the F1 and F2 generations caused by different clutch sizes^45^. In this study, in the choice test (Fig. 4d), the females had fewer offspring than females in the no-choice test (Fig. 4a–c) but also a smaller clutch size, thus ensuring a larger offspring body size and higher fertility, which increased by approximately 13 percent in the F2 generation (Fig. 4d). Other changes in the fertility of the F2 generation were not obvious (Fig. 4); however, for interest, the seemingly lower fertility in the F1 generation in relation to the fictitious host plant may ensure higher fertility in the F2 generation (Fig. 4b compared to 4a).

Interpretation of our results in the natural environment will be difficult because in nature, the offspring of switched hosts can use toxins from new host plants for their own protection^66,67^. In our experiments, the native host consumed only their native host plants; however, interestingly, with the simulation of switched hosts, the wasps with choice between the host plants had a lower fitness. It can be assumed that lower parasitoid fitness can be compensated by possible lower predation of fictitious hosts^68^. The switched host usually escapes the strong pressure of predators and parasitoids on their native plant^9,10^. Although under certain conditions, the predator avoids parasitized prey^69^, generally the parasitoid fitness is reduced more than predator fitness by intraguild predation^70,71^. Thus, for parasitoids, specialization on switched hosts should be stronger and more advantageous than for predators^12^.

**Figure 5:**
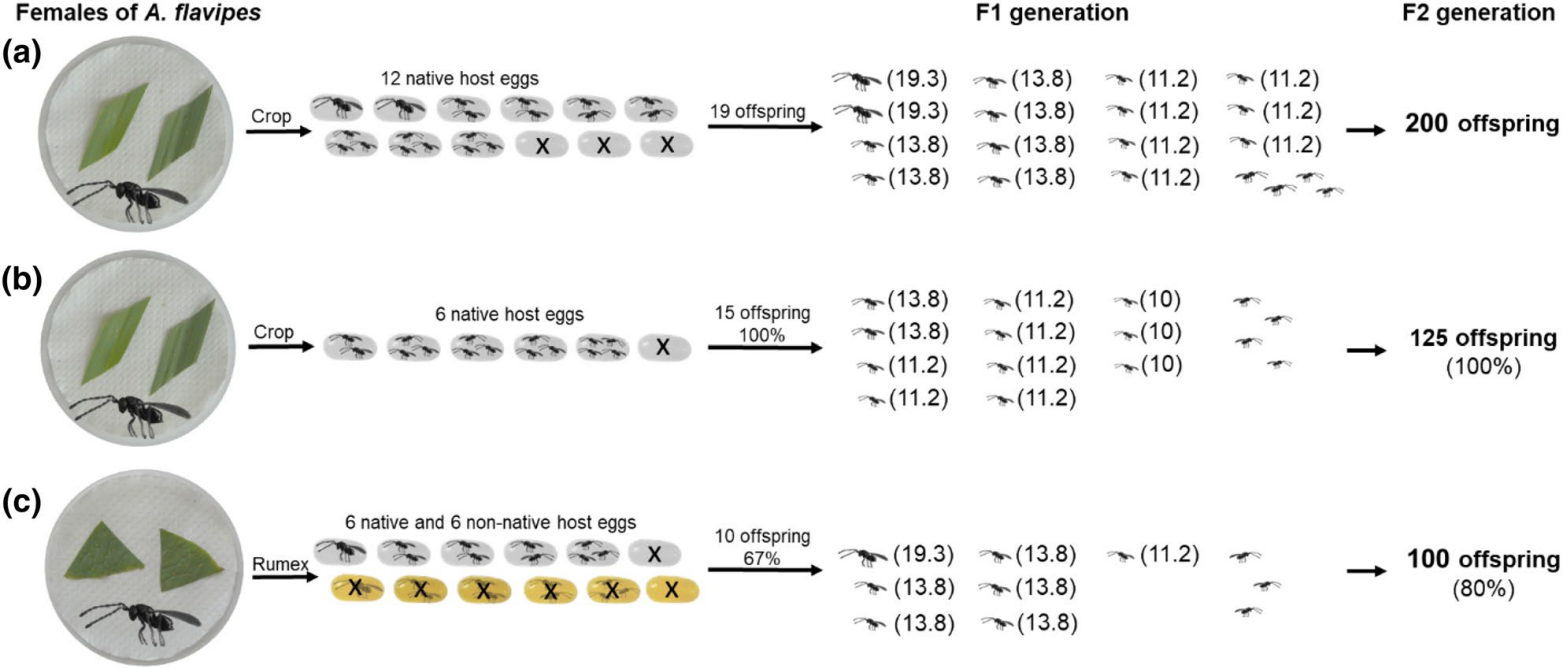
The relationship between different fitness characteristics in F1 generation and fertility in F2 generation for three groups of females in relation to occurrence of fictitious host in new environment of fictitious host plant; (**a**) females with 12 available native host on their native host plant (crop); (**b**) females with 6 available native host on their native host plant (crop) and (**b**) females with 6 available native host and 6 available fictitious host on the fictitious host plant (*Rumex*). (*The fertility values (shown in parentheses); The offspring sex ratio of *A. flavipes* is 3:1 (male:female)^46^; females are shown as (left oriented) and males as (right oriented).

Following host switching in a new environment, parasitoids can adapt to new potential host species^4^. In the second part of this study, we simulate the impact of a fictitious host on the fitness of parasitoids when they follow a switched host into a new environment. In the new environment of the fictitious host plant, the *A. flavipes* females parasitize the native host as well as the fictitious host. By parasitism, they reduce the fertility of the native and fictitious hosts and, concurrently, partly their own fertility because they were not able to complete larval development in the fictitious host (same as in our previous study Samková et al.^45^). Here, we show that the fertility of switched parasitoids is lower (females have 6 native and 6 fictitious hosts on the fictitious host plant; Fig. 5c) than the fertility of parasitoids on the usual host plant, which has high (12 native hosts; Fig. 5a) or low (6 native hosts; Fig. 5b) host population density. However, from a two-generation approach, if switched parasitoid females distribute their offspring between both the native and fictitious hosts, the clutch size decreases, and the offspring in the native host obtain a larger body size and higher fertility in the F2 generation (Fig. 5; similarly as in Samková et al.^45^). It can be assumed that in the new environment of a new host plant, among other factors^16,55^, parasitoid fitness will be reduced by parasitism of fictitious hosts in which the parasitoid is unable to complete larval development, but using this ovipositing behaviour, gregarious parasitoids increase the fertility of offspring developed in native hosts (Fig. 5; Samková et al.^45^). In addition to higher fertility, larger female offspring also obtain more females in the F3 generation offspring compared to smaller females^72^, and it can also be assumed that there is higher flight efficiency^40,73^, longevity^74^ or longer egg-laying time^75^. Additionally, by parasitizing the fictitious host, parasitoids gain the opportunity for specialization on new potential host species.

## Material and methods

### Parasitic wasps

*Anaphes flavipes* was reared from host eggs (*Oulema* spp.) collected in cereal fields in Prague (50.136° N, 14.363° E) from the end of April until the end of June in 2019. The parasitized host eggs were stored in Petri dishes with only moistened filter papers without host plants until adult wasps emerged. These “wild” wasps were used as an initial population from which the next generations of parasitoids were reared in an environmental chamber at 22 ± 2 °C with 40–60% relative humidity and continuous illumination. Subsequent generations of females and males were used for experiments. All wasps used in the experiment were naive to the host plant, that is, they were kept in Petri dishes without a host plant only on filter paper. Mated females (not older than 24 h postemergence) were placed in Petri dishes with host eggs. The females were not fed before the start of the experiment or during the experiment, and they had free access to water (modified from Samkova et al.^20,41,45,72^).

### Host species

The fictitious host species *Gastrophysa viridula* (DeGeer, 1775) (Coleoptera: Chrysomelidae) and the habitual/common hosts of the *Oulema* species complex (including two very ecologically close species, *O. duftschmidi* (Redtenbacher, 1874) and *O. melanopus* (Linnaeus, 1758) (Coleoptera: Chrysomelidae)) were used; these species were used identically as in our previous studies e.g.^20,41^ and in other studies^46,76^ because they were determined only on the basis of genital preparation^77^. In the current study, the host culture was established from adults collected in Prague (50.136° N, 14.363° E) and in Police n/Met (50.527° N, 16.245° E). The adults were kept in plastic boxes with moistened filter papers, were fed of their native host plant (*G. viridula* were fed by *R. obtusifolius* and *Oulema* spp. were fed by *T. aestivum*), and had unlimited access to water. They were allowed to lay their eggs on leaves at 22 ± 2 °C, a relative humidity of 40–60% and a 16:8-h L:D cycle. We used host eggs no older than 24 h (modified from Samkova et al.^20,41,45,72^).

### Host plant

The substrates on which host eggs were offered for parasitization were as follows: the usual host plant was *Triticum aestivum* L. (Poaceae), and *Rumex obtusifolius* L. (Polygonaceae) was used as the fictitious host plant. The usual host plant is a typical plant on which the host *Oulema* spp. occurs naturally in Europe and North America^77^, and the parasitoid *Anaphes flavipes* also occurs in this environment^46^. In the Czech Republic, *R. obtusifolius*, a plant host of *G. viridula*, which is ecologically and phylogenetically close to *Oulema* spp. is found near the land of this native plant^(pers. obs.)^, so this plant was chosen as a fictitious plant because there is a possibility that the parasitoid of *A. flavipes* will encounter this plant. The host plants were obtained at localities in Prague (50.1367° N, 14.3638° E), and they were used within three hours of collection; prior to their use, they were stored in water. The host eggs were offered to wasps for parasitization on one (no-choice experiment) or two pieces (choice experiment) of the same leaf size. As a control, the host eggs were laid loosely on filter paper.

List of terms used in the experiments:

1. Native host = *Oulema* species; native host for parasitoids *A. flavipes.*

2. Fictitious host = *Gastrophysa viridula*; non-native host for *A. flavipes.*

3. Usual host plant = *Triticum aestivum;* native host plants; it is native host plant for *Oulema* species respectively for native host of *A. flavipes.*

4. Fictitious host plant = *Rumex obtusifolius* = non-native host plants; it is native host plants for *G. viridula* respectively for non-native host of *A. flavipes.*

#### Laboratory experiments

All laboratory experiments were performed in Petri dishes (8.5 cm) in a thermal cabinet at 22 ± 2 °C and 40–60% relative humidity under a 16:8 h (L:D) photoperiod. Individual parasitized host eggs were moved to 1.5-ml plastic tubes on the 9th or 10th day after parasitization and stored at the same temperature in a thermal cabinet. The number and sex ratio of the wasps that emerged from each parasitized host egg were measured (modified from Samkova et al.^20,41,45,72^).

#### Experimental design

1. *No-choice test of host plants*
  Twelve host eggs of *Oulema* sp. were offered to each of the 60 female wasps for 8 h in three groups: the host eggs were offered (1) on the usual host plant–crop (*T. aestivum*) (n = 20); (2) on the fictitious host plant (*R. obtusifolius*) (n = 20) and (3) on filter paper (n = 20).
2. *Choice test of host plants*
  Each female (n = 20) had 12 host eggs available for parasitization for 8 h on the three different substrates in the same Petri dish (4 host eggs on the usual host plant–crop (*T. aestivum*), 4 host eggs on the fictitious host plant (*R. obtusifolius*) and 4 host eggs on filter paper).
3. *Effect of fictitious host*
  Each female had host eggs offered for parasitization in three groups: (1) simulation of switched host to the fictitious host plant (6 native and 6 fictitious host eggs on the fictitious host plant; n = 15); (2) simulation of native environment with a low population density of host (6 native host eggs on the usual host plant; n = 11); and (3) simulation of native environment with a high population density of host (12 native host eggs on the usual host plant; n = 19). For fictitious hosts, we measured the rate of emergence in (1) experiments with native and fictitious hosts (n = 114), and for controls, we measured the rate of emergence for fictitious hosts without parasitoids (n = 100).

#### Simulation of novel two-generation reproductive approach

In our previous study, we have shown that with higher clutch size the body size of the *A. flavipes* offspring decreases and at the same time, that the offspring body size determines their fertility^41^. Therefore, in the study by Samková et al.^45^ we proposed a two-generation model to see intergenerational changes in fertility when clutch size changes in the F1 generation. In this study, we applied this model to the groups of experiments where there was a change in clutch size in the F1 generation, respectively a change in offspring body size—Figs. 3, 4 and 5. In these figures, the distribution of offspring in host eggs (number and sex ratio) was determined using the frequencies of each clutch size in real data (Suppl. Mat. 4). Fertility of hypothetical group in Fig. 3 was extrapolared by formula “fertility = 20—sqrt(clutch size)“, which fitted means of all measured fertility levels (rounded to the nearest integer).

The sex ratio of offspring did not differ significantly between groups (see Results), hence the 3:1 ratio used by Anderson & Paschke^46^ in the figures (Figs. 3, 4 and 5).

### Statistical data processing

All statistical analyses were performed in R 4.0.3 (R foundation for statistical computing 2020^78^). The models used were specific for each dependent variable. For the number of offspring per dish, we used linear models (LM), and for the rate of parasitized eggs, we used generalized linear models for binomial distribution (GLM-b). The offspring sex ratio was analysed by means of mixed-effect generalized linear models for binomial distribution (GLMM-b), and clutch size was analysed either by mixed effect linear models (LMM) or by mixed-effect generalized linear models for Poisson distribution (GLMM-p, data transformation: x-1) depending on the distribution of a particular dataset. An ID specific for each dish was used as a random factor in all mixed-effect models. The mixed-effect models were built in the R package lme4^79^ and analysed by their comparison with simpler models (see below) by means of ANOVA.

Basic models always included a single fixed factor. The factors were egg substrate (*Triticum*/*Rumex*/control filter paper), substrates per dish (single substrate/mixed), or presence of leaf bug eggs (yes/no). Basic mixed-effect models were compared with null models that included the random factor only. Models assessing interactive effects between factors (e.g., different effects of substates in single-substrate and mixed dishes) always included two factors and their interaction. Mixed-effect models with interactions were compared with a model including the same two factors but not the interaction.

We calculated 95% confidence intervals for normally distributed data in R 4.0.3 (R foundation for statistical computing 2020^78^). For binomial and Poisson-distributed data, 95% confidence intervals were calculated using the R packages Hmisc^80^ and DescTools^81^, respectively.

### Ethical approval

The use of plants in the present study complies with international, national and/or institutional guidelines. The hosts plants were obtained at the same localities as hosts and parasitoids species localities in the Czech Republic. The plant samples used in the experiment are stored as dry material at the Department of Plant Protection, Faculty of Agrobiology, Food and Natural Resources, Czech University of Life Sciences Prague, Kamýcká 129, CZ-165 00, Prague 6 – Suchdol, Czech Republic: *Triticum aestivum* L. (Poaceae) and *Rumex obtusifolius* L. (Polygonaceae), CZ, Praha—Suchdol, hand collection, 1.v.2019–30.vi.2019, A. Samkova lgt., 50.1367° N, 14.3638° E, A. Samkova det. These same plant species (determined by A. Samkova) from the same host and parasitoid locations (50.136° N, 14.363° E; 50.527° N, 16.245° E) were also used for host feeding.

## Supplementary Information

Supplementary Information 1.

Supplementary Information 2.

Supplementary Information 3.

Supplementary Information 4.
